# South West Orthopaedic Club

**Published:** 1992-08

**Authors:** 


### South West Orthopaedic Club

### Meeting held at Bath Spa Hotel, 9 May 1992

**The Spring meeting of the Club was held in Bath on Saturday 9th May, 1992. The morning session consisted of an X-Ray Quiz, the demonstration of some varied and excellent clinical cases and a discussion of those cases. The afternoon session consisted of clinical papers, Abstracts which are detailed below. In these Abstracts the first Author was the Author that read the Paper.**

**Club dinner was held at the Bath Spa Hotel and a reception was held by Alex and Alistair Ross at West Combe House on Sunday lunchtime.**

**A Guest Lecture was given by Mr. John Kirkup which was entitled "The origins of Orthopaedy and the Orthopaedic Tree".**

**The Prize for the best scientific Paper was awarded to Mr Colin Dent of Swansea and Cardiff.**

### ORTHOPAEDICS IN ETHIOPIA

R. Merryweather, F.R.C.S.

This paper will describe the setting up of the first Teaching Department for Orthopaedic surgery in this fascinating country.

The idea developed from a request by World Orthopaedic Concern (W.O.C.) to explore the possibilities of Orthopaedic training in Ethiopia.

British Council in Addis Ababa gave every support, but the actual funding came from the Overseas Development Administration (O.D.A.).

The department started to function in 1987 at the height of the civil war. The first three trainees passed their Final Examination in 1991, and at this point O.D.A. support was withdrawn, so that future funding is a problem, especially as there are 12 or 15 trainees in the pipeline, at various stages.

W.O.C. is trying to make provision for this, but requires more general support both in manpower and finance.

Volunteers for overseas work with W.O.C. will find it a rewarding and educational experience whatever their status, from Senior Registrars to retired Consultants.

### OUR EXPERIENCE OF ELASTIC CEMENTLESS ACETABULAR CUPS

Dr. A. Dambreville(Quimper - France), Dr. C. Louis (Paris)

### The Implant and the Surgical Technique

Atlas is a cementless, hemispherical, elastic actebular cup, designed to be impacted. In the begining of our experience, the implant was always fixed by screws. Now a screwless fixation by "press fit" is used facilitated by its elasticity. For that we impact a bigger Atlas than the reaming. Positioning and primary stability are per-operatively controlled by testing.

### The Cases

863 cases from 1987 to 1991. The first 100 cases with 4.5 to 5 years follow up were studied. The Harris score is used for controls.

### The Results

We observed no prosthetic failure in the series of the first 100 cases. Clinical results depend more on the pre-operative states than on the type of implant: We have a high rate of congenital dislocations. Among the 863 Atlas implanted from 1987 to 1991, 9 failures were observed: 5 revisions for failed total hip replacement 2 congenital dislocations 1 Rapid Destructive Osteoarthritis and 1 protrusio acetabulum.

### Conclusion

In all failures a technical mistake could be traced: secondary stabilisation is not possible if the acetabular cup is only on bone grafts, that is to say devascularized bone. For that reason, we now use, for revisions, an Atlas cup of greater dimensions (up to 68 mm) with sound bone bearing. The grafts only fill up the lack of bony substance.

### THE EFFECT OF SURGICAL TECHNIOUE ON THE QUALITY OF HIP ARTHROPLASTY

A. J. Timperley, M.B., Ch.B., F.R.C.S., Ed.
Princess Elizabeth Orthopaedic Hospital
Wonford Road Exeter
P. R. Jones, M.Sc., Ph.D.
Department Medical Biophysics
Manchester University
R. S. M. Ling, O.B.E., F.R.C.S. Ed.(Hon), F.R.C.S.
Princess Elizabeth Orthopaedic Hospital Wonford Road Exeter

Since the operation of cemented total hip arthroplasty was described by Sir John Charnley, many changes to the original technique have been advocated. Most of the proposed changes have been supported by clinical evidence although some have gained popularity as a result of in vitro tests or on theoretical arguments. They have not all been shown to be of value in clinical practice.

This study examined the effect of alterations in surgical procedure on the clinical results and radiographic appearance of total hip replacements. On the femoral side, details of cement usage and the effectiveness of intramedullary pressurisation were correlated with results. In the acetabulum, details of bony preparation were considered as well as the duration of application of pressure to the cement.

Two sequential series of patients were examined. Data was collected on all primary Exeter total hip arthroplasties performed in 1977 and 1983 by a single surgeon. Details of the techniques employed were entered onto a proforma at the time of operation. By examining the work of 8 single surgeon other aspects of the operative procedure were kept consistent.

Complete records were available on 33 patients from the first series and 39 patients from the more recent group. The length of follow-up varied between 4 and 6 years, further follow-up to 11 year being available on patient in the first series.

X-ray analysis demonstrated significantly improved post-operative appearances when a cement gun was used in conjunction with a plug and a proximal femoral seal. There was judged to be a complete cement mantle in 92% of the later series compared with 61% of those performed in 1977. There was no case of endosteal bone lysis or cement fracture when later techniques were employed. 7% of the early group had evidence of lysis and in 17% there was a fracture of the cement mantle. There were significantly fewer lucent lines in the later series.

The use of contemporary techniques in the acetabulum also showed improved clinical and radiological results. Postoperatively 85% of cups in the later series were entirely free of radiolucent lines compared with 59% of those operations performed in 1977. By five years, 10% of arthroplasties performed in 1983 showed demarcation in all of the three zones described by DeLee and Charnley. The comparative figure for those implanted in 1977 was 17%. Over twice as many cups had migrated at five years in the earlier group.

Contemporary techniques for pressurising cement in the femur and the socket are not universally employed by orthopaedic surgeons. This prospective study demonstrates a significant improvement in the radiological result at five years if effective methods are used.

### SYNOVIAL PLICA OF THE KNEE; FACT OR FICTION

D. P. Johnson, D. M. Eastwood, P. J. Witherow
Royal Hospital for Sick Children
University Dept. of Orthopaedics, Bristol

A controlled and randomised study was performed in adolescent patients with anterior knee pain. A series of patients were selected in whom palpable tender plica were present or the plica syndrome was suspected from their symptomatology. After a period of 3 months physiotherapy and vastus medialis training, 46 cases were resistant to conservative measures. Arthroscopy was performed for each case the presence of synovial plica or other pathology was noted.

In 46 knees synovial plica were present without any other complicating pathology. The patients were then randomised to receive only diagnostic arthroscopy or arthroscopic division of all plica present. The patients were all discharged as day cases, and reviewed at 6 months, 1 year and 2 years.

The results demonstrated that there was an improvement in only 27% of patients in whom plica were present but not divided, whereas improvement of the symptoms occurred in 92% of patients in whom arthroscopic division of the plica was undertaken ($p<O.Ol$). Of the 22 cases in which arthroscopic division was not undertaken, 11 cases (50%) required further arthroscopic surgery within the two year period for severe symptoms. After arthroscopic division 8 cases improved and a good or excellent result was obtained. In 8 patients in whom both knees were affected arthroscopic division was undertaken in one knee and only diagnostic arthroscopy in the other knee. In each patient at review the knee in which division of plica was undertaken was the better knee.

The conclusion is made that synovial plica of the knee can be pathological, and is one of the many causes of anterior knee pain. Recognition of the specific symptomatology and palpation of the plica is an accurate indicator to the presence of plica and is associated with a good result from arthroscopic division. Though superior plica are the most common, the medial and lateral shelf is not uncommon. As mixed results were obtained from the anterior plica in isolation no conclusion can be made about it's pathergenicity.

### THE INFLUENCE OF THE CEMENT MANTLE THICKNESS ON FEMORAL REMODELLING FOLLOWING HIP ARTHROPLASTY

M. F. Pearse, T, H. Karachalios, V. G. Langkamer,
R. M. Atkins, L. Solomon
University Department of Orthopaedics
Bristol Royal Infirmary Bristol BS2 8HW

Marked femoral cortical hypertrophy has been observed in patients after hip arthroplasty with an uncemented porous-coated cup and cemented femoral stem. The clinical and radiographic characteristics of this phenomenon have been studied in 18 patients and compared to a randomly selected control group following similar arthroplasty without hypertrophy. Clinical outcome was assessed using the Harris hip score. The calcar width and Horsman's femoral index were measured on preoperative radiographs. The thickness of the cement mantle and extent of calcar resorption were also assessed. Both groups were evenly matched for sex, weight, Charnley functional class and for period of follow-up (mean 3.5 years). Patients with femoral hypertrophy were significantly younger than controls ($p = 0.03$). Although there was no difference between the postoperative pain scores, patients with hypertrophy had a significantly higher Harris hip score ($p = 0.02$). The cement mantle was significantly thinner and calcar resorption significantly more extensive in patients with femoral hypertrophy ($p < 0.001$). There was no difference between the calcar widths or Horsman's indices of either group.

The combination of marked cortical hypertrophy and calcar resorption suggests abnormal loading and stress shielding. These features may be predictive of early stem failure. Our results highlight the importance of the cement mantle in the normal distribution of stresses following hip arthroplasty.

### FEMORAL INTRAMEDULLARY NAILING

**Biomechanical and Clnical Considerations**

M. I. Goodwin, F.R.C.S.
Senior Orthopaedic Registrar,
The Royal United Hospital, Bath
Andrew Feben
School of Mechanical Engineering,
The University of Bath, Bath
Tony Miles, B.Sc.(Eng), M.Sc(Eng), M.B.E.S.
Lecturer in Design, School of Mechanical Engineering
University of Bath, Bath

Three basic designs of femoral interlocking nails are currently available. They differ by being of open (AO), semi-open (Grosse-Kempf) or closed (Russell-Taylor) cross-section. Good clinical results have been achieved with each product but repeated accurate distal targeting is difficult to achieve because of torsion of the nail in the distal part of the femur. In this study we have compared the mechanical characteristics of each nail to look for differences in their properties which may make distal targeting easier.

The bending stiffness and torsional rigidity were determined for three nails of each design and were correlated with theoretical data. The torsional rigidity of the Russell-Taylor, G-K and AO femoral nails measured 67, 2 and 1.4 $Nm^2$ respectively. The bending stiffness measured 128, 116 and 75 $Nm^2$ respectively. The torsional rigidity of the Russell-Taylor device was at least 30 times that of the other nails.

These results show that while the bending stiffness differs little between each design, the Russell-Taylor nail will undergo less torsional deformation when it is implanted, which may facilitate distal targeting.

### ALTERED EXPRESSION OF GLYCOSAMINOGLYCAN EPITOPES IN ACUTE KNEE JOINT TRAUMA SYNOVIAL FLUIDS

C. Dent
Department of Orthopaedic and Trauma surgery
University of Wales College of Medicine, Cardiff
P. Hazell, M. Bayliss, T. Hardingham
Kennedy Institute of Rheumatology
Hammersmith, London W6 7DW

a) Chondrocytes are responsible for the initiation of cartilage repair and remodelling processes which may occur in diseases such as Osteo- and Rheumatoid arthritis, as well as acute trauma. Such conditions may induce both quantitative and qualitative changes in proteoglycan synthesis which may manifest as alterations in proteoglycan fragments present in the joint fluid.

b) In this study we have used well characterised monoclonal antibodies, raised against carbohydrate components of proteoglycans, to detect alterations in proteoglycan fragments in synovial fluids from patients with normal and traumatised knee joints. ELISA methods were used to assay epitope levels and these were related to the total sulphated glycosaminoglycan content in the fluids.

c) Levels of the epitope recognised by M0225 (a 2,6-sulphated disaccharide unit of chondroitin sulphate) were found to be significantly higher in synovial fluids from traumatised knees. Significantly increased levels of the 4C3 epitope (a native oversulphated chondroitin sulphate isomer) and the 3D5 epitope (non-reducing terminus of chondroitin sulphate containing Hexuronic acid) were also measured in these joint fluids.

d) These epitopes may be useful as specific markers for following the progression of joint disease or for monitoring changes occurring as a result of acute joint trauma. They may also have a role in measuring the efficacy of certain treatments used following specific knee injuries which may reduce the risk of long term degenerative change.

### THE MULLER STRAIGHT-STEM FEMORAL PROSTHESIS: SEVEN YEARS ON

M. Bishay, G. Kyritsis, D. S. I. Sweetnam, A. C. Ross
Bath and Wessex Orthopaedic Research Unit
Royal United Hospital, Bath

The Muller straight-stem femoral prosthesis was introduced into the UK eight years ago. Despite being widely used, there has been no published long term follow-up study.

We retrospectively reviewed 149 consecutive patients with a minimum seven year follow-up. 33 were dead and 15 failed to attend for followup, leaving a cohort of 101 patients with 116 primary total hip replacements. These were reviewed at a research clinic with new radiographs using a standard proforma recommended by the Hip Society (1990).

Twenty-one stems (17%) had loosened radiologically: nineteen aseptically and two with sepsis. No antibiotic prophylaxis, laminar flow or cement pressurisation was routinely used at the time of implantation.

29 femora (25%) showed radiologic calcar resorption either in height or thickness, 35 (30%) femoral shafts showed evidence of stress shielding. This was defined by shaft hypertrophy at and below a point of press-fit of the stem, together with resorption of the shaft above.

Stress shielding seemed to be related to an over-sized prosthesis fitted tightly in the femoral canal. Although those who have stress shielded did not show evidence of loosening, the degree of resorption was alarming.

There has been 88 (76%) patients that had-excellent or good results, 20 (17%) had fair results and 8 (7%) had poor results.

*N.B. In all these extracts the first named author was the author that read the Paper.*

